# Role of Resolvins in the Inflammatory Resolution of Neurological Diseases

**DOI:** 10.3389/fphar.2020.00612

**Published:** 2020-05-08

**Authors:** Chunrong Li, Xiujuan Wu, Shan Liu, Donghui Shen, Jie Zhu, Kangding Liu

**Affiliations:** ^1^Neuroscience Center, Department of Neurology, The First Hospital of Jilin University, Jilin University, Changchun, China; ^2^Division of Neurogeriatrics, Department of Neurobiology, Care Sciences and Society, Karolinska Institutet, Karolinska University Hospital Solna, Stockholm, Sweden

**Keywords:** resolvins, inflammation, neurological diseases, neurodegenerative disorders, neuroimmune diseases

## Abstract

The occurrence of neurological diseases including neurodegenerative disorders, neuroimmune diseases, and cerebrovascular disorders is closely related to neuroinflammation. Inflammation is a response against infection or injury. Genetic abnormalities, the aging process, or environmental factors can lead to dysregulation of the inflammatory response. Our immune system can cause massive damage when the inflammatory response becomes dysregulated. Inflammatory resolution is an effective process that terminates the inflammatory response to maintain health. Eicosapentaenoic acid (EPA) and docosahexaenoic acid (DHA) are omega-three polyunsaturated fatty acids that play a crucial regulatory role in the development of inflammation. Resolvins (Rvs) derived from EPA and DHA constitute the Rvs E and Rvs D series, respectively. Numerous studies on the effect of Rvs over inflammation using animal models reveal that they have both anti-inflammatory and pro-resolving capabilities. Here, we review the current knowledge on the classification, biosynthesis, receptors, mechanisms of action, and role of Rvs in neurological diseases.

## Introduction

Neuroinflammation is recognized as the inflammatory reaction occurring in the central nervous system (CNS) and the peripheral nervous system (PNS), primarily caused by toxic chemicals, environmental factors, trauma, and autoimmune responses, among other factors (Refolo and Stefanova, 2019). Numerous studies have also found that the occurrence of various neurological diseases is closely related to neuroinflammation (Yuan et al., 2019). In the past, the resolution of inflammation was considered to be a passive event. Contrary to previous considerations, the peak of the acute inflammatory response is now deemed the beginning of resolution (Serhan and Levy, 2018). Resolution is an effective process that terminates the inflammatory response to maintain health. Eicosapentaenoic acid (EPA) and docosahexaenoic acid (DHA) are important omega-three polyunsaturated fatty acids (ω-3 PUFAs) that play a pivotal regulatory role in the resolution of inflammation (Joffre et al., 2019). Resolvins (Rvs), biosynthesized from essential ω-3 PUFAs EPA and DHA as precursors, are thought to have more potent anti-inflammatory and pro-resolving actions than EPA and DHA themselves (Serhan et al., 2019). Studies have revealed that Rvs exert anti-inflammatory effects in animal models of acute kidney injury, acute lung injury, ulcerative colitis, and neurodegenerative diseases, providing a new approach for the treatment of inflammatory diseases (Bento et al., 2011; Wang et al., 2011; Kaye et al., 2019). To date, there have been numerous studies on Rvs, but few comprehensive reviews of Rvs have been published. To provide an intensive and comprehensive understanding of Rvs, here, we summarized the classification, biosynthesis, receptors, mechanisms of actions, and roles of Rvs in related neurological diseases.

## Resolvins: Metabolism and Receptors

Specialized pro-resolving lipid mediators (SPMs) are part of a large family of pro-resolving molecules. Arachidonic acid-derived lipoxins (LXA4 and LXB4), EPA-derived E-series Rvs (RvE1-RvE3), DHA-derived D-series Rvs (RvD1-RvD6), protectins, neuroprotectins (PD1/NPD1 and PDX), maresins (MaR1 and MaR2), and docosapentaenoic acid (DPA)-derived 13-series Rvs (RvT1-RvT4) and their aspirin-triggered epimeric forms (AT-RvD1-AT-RvD6), are SPMs derived from PUFAs, such as ω-3 PUFAs and ω-6 PUFAs, present in dietary sources (Serhan et al., 2015; Fredman and Spite, 2017). Prostaglandins, leukotrienes, and thromboxanes also derive from arachidonic acid, an ω-6 PUFAs (Serhan et al., 2015).

SPMs are enzymatically produced in human body fluids and organs, such as peripheral blood, cerebrospinal fluid, placenta, synovial fluids, urine, sputum, spleen, lymph nodes, and others (Shang et al., 2019). The levels of EPA and DHA in the brain are mainly maintained by uptaking from dietary and/or liver sources in plasma instead of an endogenous biosynthesis (Dyall, 2015). Electrical stimulation of PNS, such as vagus, also promotes SPMs production (Serhan et al., 2018). There are two ways to biosynthesize Rvs, the lipoxygenase (LOX) mechanism and the aspirin-triggered cyclooxygenase-2 (COX-2) pathway. In vivo, the biosynthesis of RvD1 and RvD2 requires the catalysis of 15-lipoxygenase (15-LOX) and 5-lipoxygenase (5-LOX) (Videla et al., 2019). These steps can occur in mononuclear cells, such as neutrophils and macrophages, and between cells, such as leukocytes-endothelial cells and neutrophils-macrophages. The synthetic pathway of RvD3-RvD6 has yet to be reported. Additionally, RvDs are also biosynthesized through aspirin-acetylated COX-2 and undergo similar epoxidation, lipid oxidation, and hydrolysis processes to form AT-RvDs.

**Figure 1:**
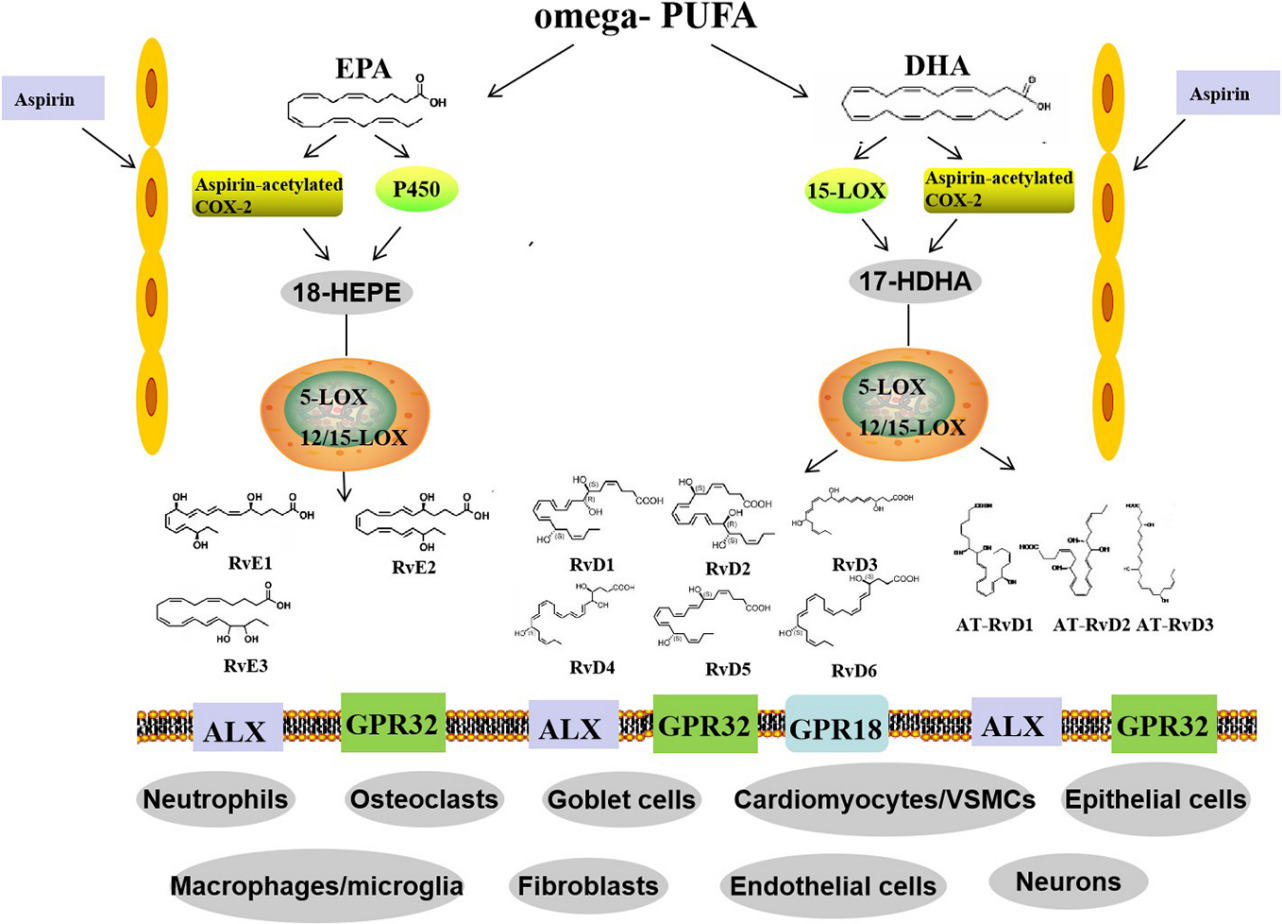
Biosynthetic routes and classification of resolvins. Omega-PUFAs include DHA and EPA. D-series resolvins derive from DHA, whereas E-series resolvins are generated from EPA. Distinct synthetic enzymes, including COX-2, cytochrome P450, 5-LOX, and 12/15-LOX are involved in these processes. Three members of the RvE family and six members of the RvD family have been identified. In addition, RvD members, called aspirin triggered RvD (AT-RvD), are also biosynthesized by aspirin-acetylated COX-2. omega-PUFA, omega-polyunsaturated fatty acid; EPA, eicosapentaenoic acid; DHA, docosahexaenoic acid; COX-2, cyclooxygenase-2; 5-LOX, 5-lipoxygenase; VSMCs, vessel smooth muscle cells.

The formation of RvE1 and RvE2 from EPA is catalyzed by aspirin-COX2 and 5-LOX through the interaction of endothelial cells and leukocytes. In addition, they can also be produced *via* an aspirin-independent pathway through the cytochrome P450-driven oxygenation of EPA (Ostermann et al., 2019). Both RvE1 and RvE2 are synthesized in response to increased 5-LOX concentrations during inflammation. However, RvE3 is different. It is generated from 18-hydroxyeicosapentaenoic acid through the 12/15-LOX pathway and can be synthesized in eosinophils (Sato et al., 2019) (**Figure 1**).

As above, Rvs are created by body’s complicated metabolic cascades. The metabolic modifications, such as oxidation, hyperoxydation, and epoxidation, are mostly unstable and susceptible to further metabolism. Indeed, Rvs are rapidly degraded or metabolized *in vivo* resulting in a short biological half-life (Valdes et al., 2017).

The pro-resolution and anti-inflammatory effects of Rvs are predominately achieved through specialized G-protein-coupled receptors (GPCRs). At present, several Rvs receptors have been identified (**Table 1**). RvD1 action is mediated by the G protein-coupled receptor GPR32 and by ALX/FPR2 (Gilligan et al., 2019). Both GPR32 and ALX are expressed in T cells and their subsets (Chiurchiù et al., 2016a; Chiurchiù et al., 2019); whereas, ALX/FPR2 is also expressed in glial cells and neurons. Furthermore, the expression of this receptor is heterogeneous and varies according to cell type, brain area, and the pathologic state of the brain tissue. These suggest that both neurons and microglia could mediate the protective effects of RvD1 *via* release of soluble factors (Krashia et al., 2019). GPR32 is abundantly expressed in macrophages, while ALX is mainly expressed in monocytes, neutrophils, and astrocytes in specific brain areas (Bisicchia et al., 2018). RvD1 can induce *de novo* regulatory T (Treg) cell differentiation mediated by GPR32 (Chiurchiù et al., 2016a). It exerts a protective effect *via* specific ALX-induced miRNA circuits to promote neuronal recovery and reduce gloss in focal brain damage models (Bisicchia et al., 2018). In addition, RvD1 provokes a reduction in the activation of microglial cells and astrocytes and prevents neuronal cell death after remote brain damage (Zhang et al., 2018). The RvD2 specific GPCR is GPR18, which is mostly expressed in monocytes, macrophages, and neutrophils (Chiang et al., 2015). Furthermore, RvD3, RvD5, and AT-RvD3 were found to bind GPR32 (Pirault and Bäck, 2018; Gilligan et al., 2019). Whereas ALX is the receptor of AT-RvD1 (Dean et al., 2019), the specific receptors for RvD4 and RvD6 remain unknown. Similarly, RvE1 exerts its biological functions through an interaction with chemokine receptor-like 1, also known as chemerin receptor 23 (ChemR23) or leukotriene B4 receptor (Oehler et al., 2017). ChemR23 is mainly expressed in monocyte cells, macrophages, microglia, and dendritic cells, mainly in CA2-4 regions and the subgranular zone of the dentate gyrus (Xu et al., 2019). ChemR23 is also detected in both neurons and glia in the human hippocampus (Wang et al., 2015). Prominently, RvD1 and RvE1 can promote resolution of inflammation in microglial cells. In vitro, ChemR23/ERV1 is expressed by retinal microglia (Chen et al., 2018). BLT1 is mainly distributed on the surface of neurons, microglia, macrophages, neutrophils, effector T cells, and dendritic cells (Ye et al., 2016). BLT1 is also an activator of ChemR23 that RvE2 can partially interact with (Deyama et al., 2018). To date, the receptor of RvE3 has not yet been identified (**Table 1**) (Pirault and Bäck, 2018).

**Table 1:** Expressions of resolvins and resolvin receptors in the central nervous system.

Subtypes	Receptors	Synthesis	Cells of the CNS	Expressions in the CNS	References
RvD1	GPR32	Catalysis of 15-COX and 5-LOX	Macrophages		(Gilligan et al., 2019; Videla et al., 2019)
	ALX	Catalysis of 15-COX and 5-LOX	Neurons, astrocytes, microglia	medial prefrontal cortex, dentate gyrus	(Bisicchia et al., 2018; Gilligan et al., 2019; Videla et al., 2019)
RvD2	GPR18	Catalysis of 15-COX and 5-LOX	Macrophages	medial prefrontal cortex, dentate gyrus	(Chiang et al., 2015; Deyama et al., 2017)
RvD3	GPR32	Not yet reported	Macrophages		(Pirault and Bäck, 2018)
RvD4		Not yet reported			
RvD5	GPR32	Not yet reported	Macrophages		(Pirault and Bäck, 2018)
RvD6		Not yet reported			
AT-RvD1	ALX	Catalysis of aspirin-LOX			(Dean et al., 2019)
AT-RvD2		Catalysis of aspirin-LOX			
AT-RvD3	GPR32	Catalysis of aspirin-LOX	Macrophages		(Gilligan et al., 2019)
AT-RvD4		Catalysis of aspirin-LOX			
AT-RvD5		Catalysis of aspirin-LOX			
AT-RvD6		Catalysis of aspirin-LOX			
RvE1	ChemR23	Catalysis of aspirin-COX2 and 5-LOX; Cytochrome P450-driven oxygenation of EPA	Neurons, microglia	hippocampus, CA2-4 regions subgranular zone of the dentate gyrus	(Wang et al., 2015; Oehler et al., 2017; Xu et al., 2019)
	BLT1	Catalysis of aspirin-COX2 and 5-LOX; Cytochrome P450-driven oxygenation of EPA	Neurons, microglia, Macrophages		(Ye et al., 2016)
RvE2	ChemR23	Catalysis of aspirin-COX2 and 5-LOX; cytochrome P450-driven oxygenation of EPA	Neurons, microglia,	hippocampus, CA2-4 regions subgranular zone of the dentate gyrus	(Deyama et al., 2018; Ostermann et al., 2019)
	BLT1		Macrophages		(Deyama et al., 2018)
RvE3		Generated from 18-HEPE through the 12/15-LOX pathway			(Sato et al., 2019)

## Mechanism of Rvs in the Resolution of Inflammation

### Mitogen-Activated Protein Kinase Signaling Pathway

The mitogen-activated protein kinase (MAPK) family is a group of serine protein kinases that can be activated by different extracellular stimuli. To date, several parallel MAPK signaling pathways, such as the c-Jun N-terminal kinase (JNK)/stress-activated protein kinase, the extracellular-signal regulated kinase (ERK), and the p38MAPK pathways, have been identified (Celis-Plá et al., 2019). The activation of the different MAPK signaling pathways depends on the stimulus. Several studies have indicated that Rvs play a role in the resolution of inflammation *via* a MAPK signaling pathway. Pretreatment with RvD1 can significantly inhibit the activation of ERK1/2, p38MAPK, and JNK in lung tissues of mice and sharply decrease the proinflammatory cytokines levels (Wang et al., 2011). Moreover, in a model of osteoarthritis induced by interleukin (IL)-1β, RvD1 can reduce the production of proinflammatory mediators by blocking the phosphorylation of p38MAPK and JNK induced by IL-1β (Benabdoune et al., 2016).

### NF-κB Signaling Pathway

NF-κB regulates the expression of tumor necrosis factor-α (TNF-α), IL-6, monocyte chemoattractant protein 1, IL-8, E-selectin, chemoattractants, and adhesion molecules (Sun et al., 2019). Rvs can inhibit the NF-κB signaling pathway and play an anti-inflammatory role; signal transduction inhibition is achieved by acting at different sites throughout the pathway. Wang et al. (2011) reported that pretreatment with RvD1 prominently inhibited I-κB activation in mice lung tissues and production of TNF-α and IL-6 in bronchoalveolar lavage fluids; besides the expression of the adhesion molecule COX-2 and the inducible nitric oxide synthase (iNOS) in lung tissues was sharply decreased (Wang et al., 2011). Treating animals with AT-RvD1, RvD2, or 17R-hydroxy docosahexaenoic acid significantly reduced NF-κB mRNA expression and protein activation compared with those expression and activation levels observed after a treatment with dextran sulfate sodium (Bento et al., 2011). It was also found that 10, 17S-docosatriene inhibited leukocyte infiltration, NF-κB, and COX-2 induction in experimental stroke and elicited neuroprotection. In vitro, this lipid messenger inhibits both IL-1β-induced NF-κB activation and COX-2 expression (Marcheselli et al., 2003). RvD1 binding to ALX/FPR2 receptor can decrease the expression levels of the NF-κB protein and increase the expression of the NF-κB inhibitor protein, effects that can be eliminated by an antagonist of the ALX/FPR2 receptor and by the action of an ALX/FPR2 siRNA. AT-RvD3 can reduce the phosphorylation of NF-κB and the expression of the proinflammatory factor IL-6, which is the proinflammatory target of pNF-κB (Colby et al., 2016). RvD2 was found to downregulate the expression of the toll-like receptor 4 (TLR4)/NF-κB p65 gene by reducing the nuclear translocation of TLR4/NF-κB pathway p65 in microglia, blocking the transmission of the NF-κB signaling pathway (Tian et al., 2015). Ishida et al. (2010) found that pretreatment with RvE1 can inhibit TNF-α-induced nuclear translocation of NF-κB in a ChemR23-dependent manner in HEK293 cells (Ishida et al., 2010). These results demonstrate that RvE1 regulates the proinflammatory responses of those macrophages that express ChemR23 (Xu et al., 2019). The effects of RvE1 on macrophages were also studied by Flesher et al. (2014); they found that the translocation of p65 protein into the nucleus was dramatically lessened after treatment with RvE1. They demonstrated that the effects of RvE1 on macrophages could be associated with inhibition of the NF-κB pathway (Flesher et al., 2014).

### Phosphatidylinositol-3-Kinase Signaling Pathway

The phosphatidylinositol-3-kinase (PI3K)/Akt signaling pathway is related to the occurrence and development of tumors, inflammation, and autoimmune diseases as it can regulate cell proliferation, differentiation, translation, and transcription. LY294002 is a PI3K-selective inhibitor that competes for the ATP-binding site of the enzyme. The cardioprotective effect of RvD1 is abrogated by LY294002, indicating that the RvD1 protective mechanism is associated to the PI3K/Akt signaling pathway activation (Gilbert et al., 2015). Similarly, in animal models of Sjogren’s syndrome, RvD1 reduced TNF-α-mediated damage during the formation of the salivary epithelium by regulating the PI3K/Akt signaling pathway (Ohira et al., 2010). RvE1 can enhance macrophage phagocytosis through the PI3K/Akt pathway. Ohira et al. (2010) have proposed that, in ovarian cells transfected with the ChemR23 receptor, RvE1 enhanced Akt phosphorylation; by using an Akt phosphorylation antibody, they showed that RvE1 affects both Akt and ribosomal protein S6 phosphorylation (Ohira et al., 2010). Both RvD1 and RvE1 have the ability to prevent murine fibroblast proliferation by reducing the activity of the Akt and ERK pathways (El Kebir et al., 2012). Binding of RvE1 to ChemR23 initiates PI3K signaling, leading to the phosphorylation of Akt and a ribosomal S6 protein, also through the mTOR signaling pathway, regulating the resolution of inflammation (Easley et al., 2016). RvE1 also enhances apoptosis in human neutrophils and suppresses murine osteoclast growth by inhibiting AKT and/or ERK phosphorylation (El Kebir et al., 2012; Jung et al., 2014).

### Other Signaling Pathways

Rvs also regulate specific microRNAs (miRNAs) and markers of apoptosis, including caspases 3/9, B-cell lymphoma-2 (Bcl2), lactic dehydrogenase (LDH), and Akt, among other intracellular mechanisms of action (Bisicchia et al., 2018). MiRNAs are known to play a significant role in cell differentiation and apoptosis, biological development, and disease occurrence. MiR-219-5p, miR-208, miR-146b, and miR-21 were the first miR signatures related to the resolution of self-limited acute inflammation (Fredman et al., 2012). This group has found that RvD1 decreases miR-219, miR-21, and miR-146b expression, whereas RvD2 decreases miR-146b and miR-21 and shows little effects on the regulation of miR-219-5p. Besides, RvE1 decreases miR-219-5p and miR-21 but does not decrease miR-146b (Ohira et al., 2010). Considering that miRNAs are involved in the inflammatory response, regulating miRNAs levels may be one of the mechanisms of Rvs inflammation regression (Fullerton and Gilroy, 2016). A report on the inhibition of apoptotic signaling by an inflammatory dissipation factor showed that RvD1 blocked caspases activation by hydroxynonenal and decreased LDH release. Furthermore, RvD1 abrogated the hydroxynonenal-induced decrease in the expression of the anti-apoptotic factor Bcl2. These findings confirm that RvD1 is an anti-apoptotic molecule, which can regulate a variety of apoptotic mediators, including caspases, LDH, and Bcl2, when added to cultured cells (Benabdoune et al., 2016). In turn, RvD2 can increase the binding of Rac to GTP and activate the Rac/eNOS pathway by binding to receptor GPR18 (Zhang et al., 2016). The mechanism of the administration of rosiglitazone is that the activation of the proliferator-activated receptor-γ (PPAR-γ) with thiazolidinedione drugs, inhibits persistent pain which was elucidated by Taylor, (2015). They proposed that the resolution of inflammation, which is disrupted in diabetes, is induced by exogenous RvD1; moreover, RvD1 can promote the PPAR-γ-mediated shift of M1 macrophages to the M2 phenotype, thereby generating analgesia (Taylor, 2015). Modulation of oxidative stress is another signaling way which Rvs exert their function. As we all know that oxidative stress plays a crucial role in the pathogenesis of chronic inflammatory disease (Chiurchiù et al., 2016b). Rvs modulate oxidative stress mainly by reducing the production of reactive oxygen and nitrogen species. In addition, Rvs can also potentiate several antioxidant defenses, such as superoxide dismutase, heme oxygenase-1, and nuclear factor-E2-related factor expression (Leuti et al., 2019).

## Roles of Rvs in Inflammatory Resolution of Neurological Diseases

### Rvs in Neurodegenerative Disorders

**Figure 2:**
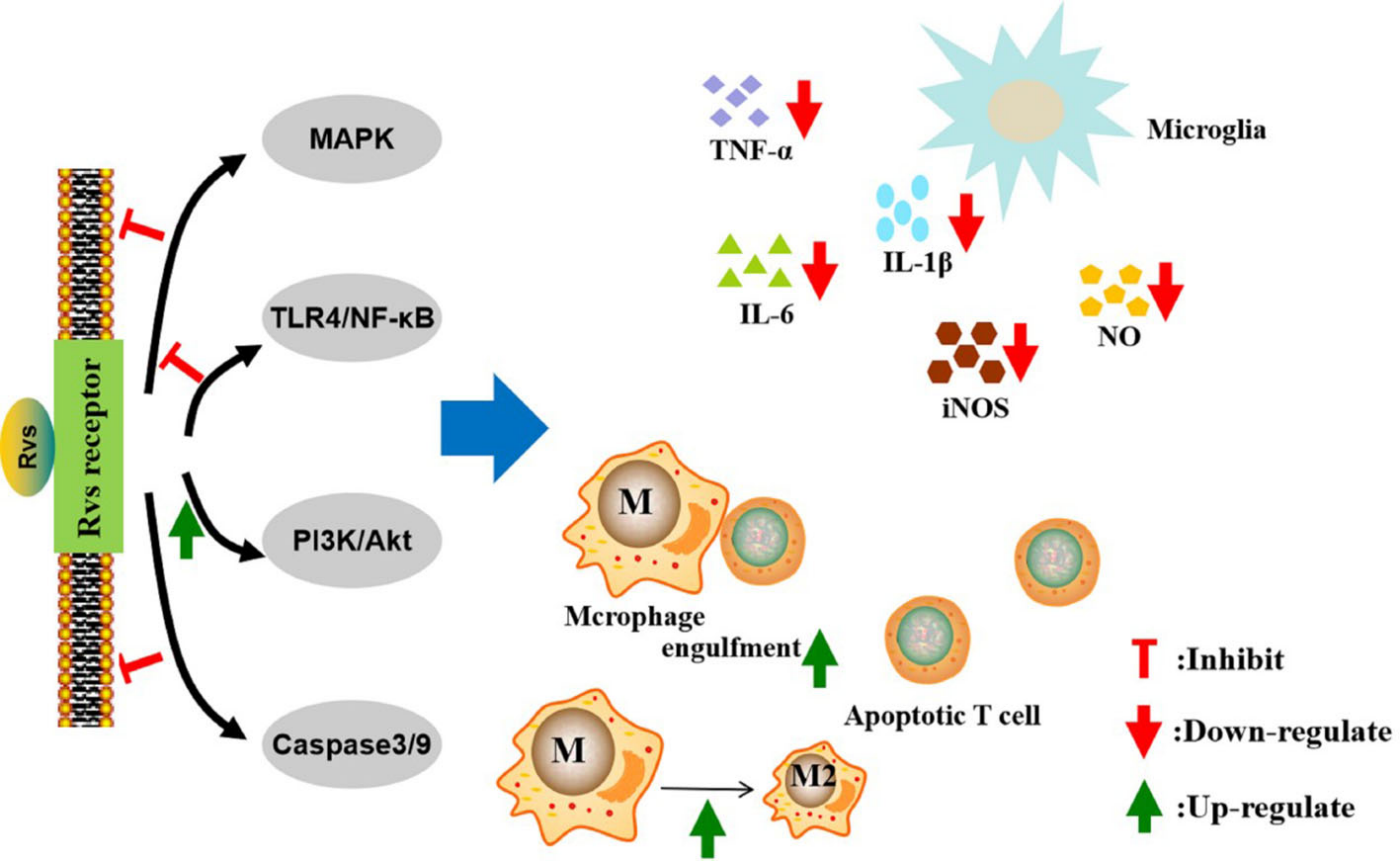
Mechanisms of action of resolvins in the resolution of neurological diseases. The pro-resolution and anti-inflammatory effects of resolvins are predominately achieved through specific G-protein coupled receptors. The activation of macroglia/macrophages is increased in neurological diseases. Resolvins can inhibit macroglia activation and reduce the proinflammatory cytokines, such as TNF-α, IL-6, IL-1β, iNOS, and nitric oxide, through the MAPK, NF-κB, PI3K/Akt, and caspase-3 signaling pathways. Resolvins can promote macrophages’ anti-inflammatory phenotype and enhance phagocytosis of Aβ and apoptotic T cells. Rvs, resolvins; MAPK, motgen-activated protein kinase; NF-κB, nuclear factor-κB; PI3K, phosphatidylinositol-3-kinase; TNF-α, tumor necrosis factor α IL-6, interleukin-6; IL-1β, interleukin- 1β; iNOS, inducible nitric oxide synthase; NO, nitric oxide.

Alzheimer’s disease (AD) is considered as the most common type of dementia; the number of AD patients is rapidly increasing. It is well known that the deposition of amyloid β (Aβ) protein in the human brain and the formation of neurofibrillary tangles, consisting of intraneuronal hyperphosphorylated tau, are the main histopathological markers of AD. In vivo positron emission tomography imaging showed increased microglial activation in AD patients, providing a direct evidence of brain inflammation (Raj et al., 2017). Moreover, levels of proinflammatory cytokines are enhanced in both postmortem brain tissue and serum from AD patients (Banerjee et al., 2017; Raj et al., 2017). Resolution is an effective process that terminates the inflammatory response to promote healing and return to homeostasis. The inflammatory resolution is an active procedure mediated by SPMs. The levels of RvD1 and LXA4 in cerebrospinal fluid (CSF) and hippocampal tissues of AD patients after death were significantly lower than those of non-AD patients; these levels showed a direct relation with the patients’ mini-mental state test scores and tau protein accumulation in the brain (Wang et al., 2015). Aβ plaques activate the brain innate immune cells and trigger their pro-inflammatory signaling pathways, which can cause increased Aβ. Alternatively, Aβ was phagocytosed by microglia (Newcombe et al., 2018). SPMs MaR1 improves neuronal survival and exerts a stimulatory effect on microglial uptake of Aβ by binding to PPAR-γ (Zhu et al., 2016). RvD1 can reduce the proinflammatory phenotype of microglia and enhance phagocytosis of Aβ by microglia of AD patients, which is consistent with data suggesting that Rvs promote clearance of Aβ deposition to reduce inflammation in AD. RvD1 ameliorates the decline of phagocytosis of FAM-Aβ through binding to different receptors. The effects *in vitro* were concentration-dependent on MAPK, PI3K, and calcium signaling pathways (Mizwicki et al., 2013). Rvs regulate the inflammatory process does not inhibit the activity of those enzymes directly, but activate specific mechanisms through receptor-ligand interactions to promote homeostasis (Mizwicki et al., 2013). Reversion of neuroinflammation is an intricate and active process, different Rvs have different selective functions and accommodate this procedure at multiple levels. Defective phagocytosis of Aβ and an abnormal activation of the inflammatory response are also crucial immune pathologies found in patients with mild cognitive impairment (MCI). A recent study also showed that increased RvD1 levels improve Aβ phagocytosis and mediate inflammatory gene expression toward a physiological state in MCI patients (Fiala et al., 2016) (**Table 2**, **Figure 2**). Kantarci et al. (2018) also found that the treatment with RvE1 and LXA4, alone or in combination, reduced Aβ plaque deposition and restored homeostasis *in vivo* reversing the inflammatory process due to inflammatory cytokines and chemokines as measured by multiplex immunoassay in 5xFAD mice (Kantarci et al., 2018). Compared to untreated 5xFAD mice, the combination of RvE1 and LXA4 also reduced the ratio of activated/total microglial cells, but neither LXA4 nor RvE1 affected the activation of microglia (Kantarci et al., 2018). BLT1 and ChemR23 are receptors of RvE1 and the levels of these two receptors are increased in the limbic system, frontal cortex and cerebellum in AD patients (Emre et al., 2020). RvE1 may decrease the release of inflammatory cytokines and resolve chronic inflammation by binding to receptors of glial and neuronal cells (Emre et al., 2020). It was also found that administration with a moderate dose of RvE1 in Ts65Dn mice (mouse model for Down syndrome related AD) reduced the levels of serum pro-inflammatory cytokines, microglial activation in the hippocampus, and the memory loss, suggesting Rvs may represent a new therapeutic target for individuals with DS and others at risk of developing AD (Hamlett et al., 2020).

**Table 2:** Roles of resolvins in the resolution of neurological diseases.

Neurological diseases	Mediators	Action	References
AD	RvE1	Decrease the levels of Aβ and restore homeostasis to reverse the inflammatory process of inflammatory cytokines and chemokines;	(Kantarci et al., 2018)
	RvE1 with LXA4	Decrease the ratio of activated/total microglia;	(Kantarci et al., 2018)
	RvD1	Reduce macrophage pro-inflammatory phenotype and enhance phagocytosis of Aβ;	Mizwicki et al., 2013)
PD	RvD2	Prohibit the activation of macroglia and reduce the activation of microglia;	(Tian et al., 2015)
	RvD2	Downregulate TNF-α, IL-1β, iNOS, NF-κ, NO, and ROS production;	(Liu et al., 2012)
ALS	RvD1	Negatively regulate chemokines, cytokines and co-stimulatory molecule;	(Correale et al., 2017b)
MS	RvD1	Reduce inflammation caused by oxidative stress, inhibit the entry of leukocyte into inflammatory sites and clear macrophages;	(Pruss et al., 2013)
	RvD1	Influence the amount of infiltrating monocytes/macrophages in the CNS, and also induces the M2 phenotype;	(Poisson et al., 2015)
	RvD1	Induction of regulatory T cell polarization of monocytes/macrophages and microglia into the M2 phenotype; Increase in myeloid-derived suppressor cells;	(Das, 2012)
GBS	RvD1	Promote macrophage phagocytosis of apoptotic T cells in the PNS, upregulate TGF-β by macrophages, increase Treg cell counts;	(Luo et al., 2016)
Ischemic stroke	RvD2	Decrease the release of TNF-α and IL-6 in the brain;	(Zuo et al., 2018)
	RvD1 and RvD2	Inhibit vascular smooth muscle cell proliferation, migration, monocyte adhesion, superoxide production, and proinflammatory gene expression;	(Miyahara et al., 2013)
	RvE1	Increase Akt, ERK1/2, and endothelial nitric oxide synthase phosphorylation and attenuate the activated caspase-3 levels and the levels of phosphorylated p38;	(Keyes et al., 2010)

Parkinson’s disease (PD) is the second most common degenerative disease of the CNS after AD. The main pathological feature of PD is the progressive degeneration of dopaminergic neurons located in the dense part of the substantia nigra, leading to decrease concentrations of dopamine in the striatum (da Cruz Moreira-Junior, 2019). Microglial cell activation is considered to play a key role in the pathogenesis of PD, by exerting toxic effects on neurons (Chen et al., 2017). Massive proinflammatory cytokines, nitric oxide, reactive oxygen metabolites, and other substances are released by activated microglial cells (Raj et al., 2017). Microglia-mediated neuroinflammation contributes to the cascade of events that lead to degeneration and worsening of PD. Krashia et al. (2019) have found that altered dopamine neuron properties, reduced striatal dopamine outflow, and motor deficits seen in transgenic rat models of PD are coupled with microglial activation and perturbations in the inflammatory response as well as in RvD1 levels. They also found that RvD1 administration in a transgenic rat model of PD can prevent central and peripheral inflammation, neuronal dysfunction, and motor deficits. Significantly, endogenous RvD1 is decreased in early-PD patients. In general, these findings set the basis for using RvD1 as a clinical biomarker of inflammation and highlight the translational potential of RvD1 (Krashia et al., 2019). Different concentration of RvD2 were used to treat lipopolysaccharide-induced inflammatory nerve damage in rat models of PD, experimental results demonstrated that RvD2 significantly inhibited microglial activation (Tian et al., 2015). Moreover, Tian et al. (2015) also found that RvD2 inhibited NF-κB, TNF-α, IL-1, IL-18, IL-6, IL-1β, and iNOS mRNA expression in primary microglial cells *in vitro* (Tian et al., 2015). Collectively, these results suggest that RvD2 can reduce microglial cells’ inflammation by inhibiting the activation of microglial downstream TNF-α, IL-1β, and iNOS signaling and by upregulating mRNA expression along the NF-κB p65 pathway, NO release, and production of ROS (Tian et al., 2015). Therefore, RvD2 may be an innovative hotspot for treating PD (**Figure 2**).

Amyotrophic lateral sclerosis (ALS) is a rare neurodegenerative disorder of unclear origin. Nevertheless, there is evidence showing that the spinal cord is infiltrated by macrophages and T cells. In a vitro ALS model, macrophages displayed aggregations of superoxide dismutase-1 and increased expression of inflammatory cytokines, including IL-1β, IL-6, and TNF-α. Additionally, in macrophages of ALS, RvD1 showed activity against IL-6 and TNF-α. Several chemokines, including chemokine 8, chemokine 9, and chemokine 10, and the cytokine IL-17C as well as the co-stimulatory molecule CD40LG play an important role in the inflammatory activation process by attracting mast cells, monocytes, and T cells into the spinal cord of patients with ALS (Liu et al., 2012). These molecules are negatively regulated by RvD1. Furthermore, RvD1, RvD2, and MaR1 are critical in modulating T cell responses. Both RvD1 and RvD2 control CD4^+^ T cell differentiation into Th1 and Th17 effectors with decreased production of interferon-γ (IFN-γ) and IL-17 (Serhan and Levy, 2018). MaR1 can increase the proportion of Treg cells while reduce proportion of Th17 cells under specific differentiation conditions (Jin et al., 2018). Thus, Rvs provide a novel method to suppress inflammatory activation in ALS (**Table 2**, **Figure 2**).

### Rvs in Neuroimmune Diseases

Multiple sclerosis (MS) is an autoimmune disease characterized by inflammatory demyelination of white matter in the CNS. In MS, immune cells induce both demyelination and axonal damage (Correale et al., 2017a). Although its etiology remains elusive, it is now known that exogenous environmental factors and susceptible genetic factors are involved in disease pathogenesis (Ontaneda et al., 2017). Lipid mediators functioning as agonists for resolution in the CSF of MS patients with either a “high active” or a “low active” were analyzed. Results demonstrated that not only the expression of inflammatory mediators in the CSF was different, but also the synthesis of pro-resolution lipid mediators differed between “highly active” and “less active” MS patients (Pruss et al., 2013). In addition, a correlation between the severity of the disease and the resolution-promoting mediator RvD1 was identified. RvD1 has also been shown to reduce inflammation caused by oxidative stress, inhibit the entry of leukocytes, and remove macrophages from inflammatory sites (Pruss et al., 2013). The role of RvD1 was also explored in MS mouse models by Poisson et al. in 2015. Their results suggest that administration of RvD1 is effective in attenuating experimental autoimmune encephalitis (EAE) disease progression compared with the vehicle group where disease’s incidence appears unaffected. Treatment with RvD1 affects the quantity of infiltrating monocytes/macrophages in the CNS and induces the macrophage M2 phenotype (Poisson et al., 2015). Multiple mechanisms are involved in RvD1-mediated protection in EAE, including induction of Treg cells, polarity monocytes/macrophages and microglia into the M2 phenotype, and an increase in myeloid-derived suppressor cells (Das, 2012). Moreover, Rvs can help treat neuronal damage in MS and enhance the recovery process by increasing stem cell survival, proliferation, and neuronal differentiation (Das, 2012). RvD1 plays an effective role in alleviating clinical manifestations in preclinical MS mouse models (Poisson et al., 2015) (**Table 2**, **Figure 2**).

High-mobility group box 1 (HMGB1), a nuclear protein with pro-inflammatory properties, can promote the neuroinflammatory processes in MS and EAE through the positive feedback loop involving infiltrating macrophages (Andersson et al., 2008). The differentiation of monocytes into macrophages can be regulated by HMGB1 and HMGB1 plus C1q, respectively. HMGB1 induces leukotriene B4 (LTB4) production through a receptor for advanced glycation end product (RAGE)-dependent pathway and causes a positive-feedback loop between the LTB4 and IFN regulatory factor 5 (IRF5), a transcription factor critical for the induction and maintenance of proinflammatory macrophages. In contrast, HMGB1 plus C1q induces the production of SPMs LXA4, RvD1, and RvD2 by a RAGE- and leukocyte-associated Ig-like receptor-1 (LAIR-1)-dependent pathway and Rvs block IRF5 induction and prevent the differentiation of inflammatory macrophages (Liu et al., 2019). In addition, lipoxins and Rvs can also enhance macrophage phagocytosis through PKC can PI3K (Mitchell et al., 2002; Linton et al., 2016).

Numerous epidemiological studies have indicated that high-density lipoprotein (HDL) cholesterol levels were negatively correlated with atherosclerotic cardiovascular disease (ASCVD) (Huang et al., 2020). Recently several studies also reported that HDL was associated with MS progression (Gafson et al., 2018). HDL fractions from healthy volunteers limited the pro-inflammatory LTB4 production and enhanced anti-inflammatory LXB4 and RvE2 production, as well as enhanced macrophage phagocytosis, through endocytic engulfment into activated macrophages. HDL fractions from patients with recurrent coronary atherosclerotic disease released *de novo* local LTB4, which blocked endocytic engulfment of HDL by macrophages and did not show anti-inflammatory effects. These results suggest a novel mechanism of HDL regulates the 5-LOX/LTB4 pathway in macrophages (Tsuda et al., 2017). EPA-rich reconstituted HDL (rHDL) particles exhibit cardioprotective properties *via* the production of RvE3 and the increase in cholesterol effux (Tanaka et al., 2018). The increased anti-inflammatory effects of EPA-rich HDL may be responsible for EPA itself and the production of RvE3. Thus, the EPA and RvE3 are involved in the anti-inflammatory effects of HDL. These results all suggest that Rvs can now provide with novel therapeutic strategies for MS.

The Guillain-Barré syndrome (GBS) is a common acute immune-mediated inflammatory disease of the PNS, characterized by inflammatory infiltration and damage to myelin sheaths and axons (Zhang et al., 2013). The acute inflammatory demyelinating polyradiculoneuropathy (AIDP) is the most common clinical GBS subtype, whereas experimental autoimmune neuritis (EAN) is the classical animal model for AIDP (Langert et al., 2017). The induction of Treg cells and anti-inflammatory macrophages is crucial for inflammation resolution and for a spontaneous recovery of neuropathies and EAN (Shi et al., 2018; Zhang et al., 2019). Thus, phagocytosis of apoptotic T cells seems to play a significant role in autoimmune inflammation resolution and connects the known pro-resolving factors in EAN. Luo et al. (2016) found that RvD1 significantly enhanced macrophage engulfment of apoptotic T cells, decreasing apoptotic cell accumulation in the PNS of EAN rats. Moreover, they also revealed that RvD1 promotes the polarization of anti-inflammatory macrophages and induces transforming growth factor-β (TGF-β) production, leading to the inflammation resolution response in EAN. That RvD1 contributed to the induction of Treg cells in EAN was also observed (Luo et al., 2016). Moreover, accumulating evidence has revealed that Treg cells contribute to the promotion of the inflammatory resolution response in autoimmune inflammatory disorders, especially, in EAN (Shi et al., 2018). Mechanistically, RvD1-induced TGF-β promotes macrophage phagocytosis of apoptotic T cells, which contributes to an increase in Treg cells number in EAN (**Table 2**, **Figure 2**) (Luo et al., 2016). Collectively, these data suggest that RvD1 has a significant role in resolving inflammation and it may be a therapeutic target in human neuritis (Luo et al., 2016).

### Rvs in Cerebrovascular Disorders

Ischemic stroke is a worldwide heavy burden cerebrovascular disease. Cerebral ischemia/reperfusion (I/R) injury is a key factor leading to poor prognosis. Cerebral I/R injury includes increased vascular permeability, destruction of the blood-brain barrier (BBB), and brain edema (Cassella and Jagoda, 2017). Brain microvascular endothelial cells (BMVECs) are the main cells from the BBB and also the main target cells producing reactive oxygen species and inflammatory reactions during cerebral I/R (Reinhold and Rittner, 2017; Albekairi et al., 2019). It has been seen that inflammatory cytokines increase significantly after brain necrosis induced by I/R (Jiang et al., 2017). Thus, feasible therapeutic targets for ischemic stroke are BMVECs and neurons and their protection from the oxidative stress and inflammation induced by I/R (Zuo et al., 2018a). Exogenous applied RvD2 reduces the release of TNF-α and IL-6 in the brain, decreases the infarct area, and protects the neurons and BMVECs from apoptosis and necrosis after middle cerebral artery occlusion and reperfusion (Zuo et al., 2018a). Through enzymatic reactions mediated by 12/15-LOX and 5-LOX, it is possible to biosynthesize RvD1, PD1, and LXA4. These mediators regulate the magnitude of the local inflammatory response of macrophages and vascular endothelial cells by exerting a potent agonist action (Wu et al., 2017). RvD1 and RvD2 also have a dose-dependent effect in the inhibition of vascular smooth muscle cell (VSMC) proliferation, migration, monocyte adhesion, superoxide production, and the expression of proinflammatory genes. Thus, RvDs reduce early inflammation reactions and subsequent neointimal hyperplasia due to a broad inhibition of VSMC activation responses and the modulation of vascular injury responses *in vivo* (Miyahara et al., 2013). RvE1 has an immediate protective effect on myocardial cells both *in vitro* and *in vivo*.

Keyes et al. (2010) showed that, *in vivo* experiments, RvE1 mediated the reduction of a myocardial infarct area in a dose-dependent manner. In vitro, different doses of RvE1 in cells subjected to hypoxia or hypoxia/reoxygenation conditions, improved cell survival and reduced apoptosis, also in a dose-dependent manner. Further, it was observed that RvE1 can increase Akt, ERK1/2, and endothelial nitric oxide synthase phosphorylation and attenuate activated caspase-3 and phosphorylated p38 levels. In conclusion, RvE1 has a direct protective action on cardiomyocytes against I/R injury and restricts infarct area when administered intravenously before reperfusion (Keyes et al., 2010) (**Table 2**, **Figure 2**).

Rvs studies on other vascular diseases have shown that patients with symptomatic peripheral arterial disease have significantly lower plasma levels of aspirin-triggered lipoxin than healthy controls. Both aspirin-triggered lipoxin and RvE1 may hinder platelet-derived growth factor-stimulated migration of human saphenous vein vascular smooth muscle cells and decrease phosphorylation of the platelet-derived growth factor receptor-β (Ho et al., 2010). The presence of both the aspirin-triggered lipoxin and the RvE1 receptors was confirmed in human VSMCs. These results suggest that Rvs play an active role in accelerating inflammation resolution *via* altering the VSMCs phenotype and provide a potential curative chance for regulating vascular injury responses in vascular diseases (Ho et al., 2010). Clinical studies have led to the proposal that Rvs have protective effects on vessels and cardiomyocytes in patients with abdominal aortic aneurysm (Pillai et al., 2012).

## Potential Therapeutic Prospects of Rvs in Neurological Diseases

Persistent inflammation and impaired inflammatory resolution are the main constituents of neuroinflammation. Thus, the ideal drug for the treatment of neuroinflammatory diseases should inhibit inflammation and activate resolution. At present, in most animal models of neuroinflammatory diseases, it has been shown that the protective effect of Rvs can be exerted through different mechanisms. Rvs have powerful anti-inflammatory abilities, they participate in anti-inflammatory and proinflammatory subsidence by affecting signaling pathways, such as MAPK, NF-κB, PI3K, miRNAs, apoptosis, and PPAR-γ. They have shown good effects over inflammation, fibrosis, pain, metabolism, anti-depressant activities, and other diseases (Dean et al., 2019). As mentioned above, the receptors of RvD1 are abundantly expressed in macrophages, glial cells, and neurons. Moreover, RvD1 has the ability to reduce activation of microglia and astrocytes and prevent neuronal cell death after remote brain damage. Thus, we speculate that RvD1 may be one of the most interesting Rvs to continue with RvD1 for novel treatment design. But there are a number of limitations to use Rvs as therapeutic targets. First, Rvs production is deficient in certain diseases which are related to chronic, unresolved inflammation, although Rvs are generated in healthy human volunteers following a dietary replenishment of DHA or EPA (Serhan et al., 2019). Many studies in rodents and isolated human cells have unequivocally shown that the pharmacological activities of Rvs can be inactivated by local metabolic pathways and that they are difficult to produce from synthetic pathways. Second, the development of stable analogs of Rvs and the improvement of the delivery methods required to heighten and extend the pro-resolving action of Rvs is still challenging. Rvs are a kind of lipid mediators with powerful anti-inflammatory effects, and the clinical application prospect of Rvs has attracted much attention and expectation. Finally, the main challenge is the determination of the molecular mechanisms underlying the protective effect of Rvs in neurological diseases. The determination, for example, of those Rvs that are the most important for treating the different neurological diseases and the optimal routes of administration or the optimal combination of current and new therapies. Collectively, the acquisition of that knowledge that currently limits the clinical application of Rvs (Serhan et al., 2019). A thorough understanding of the protective effects and mechanisms underlying inflammation may help to develop effective methods for the treatment of various neuroinflammatory diseases.

Noteworthy, both RvD1 and RvD2 can act on brain regions, such as the medial prefrontal cortex and the dentate gyrus, to exert antidepressant effects *via* the mammalian target of rapamycin complex 1 signaling pathway (Deyama et al., 2017).

## Conclusions

Neuroinflammation is an inflammatory response occurring both in the CNS and the PNS; it is involved in a complex cascade of proinflammatory and anti-inflammatory signaling events and constitutes a central process in many neurological diseases. Rvs are effective compounds that terminate the inflammatory response to maintain health. Rvs exert anti-inflammatory and proinflammatory subsidence effects by affecting signaling pathways, such as MAPK, NF-κB, PI3K, miRNAs, apoptosis, and PPAR-γ. Rvs show great anti-inflammatory effects in neurodegenerative diseases, neuroimmune diseases, and cerebrovascular disorders, thus providing a new way of thinking for the treatment and prevention of neurological diseases. There are, nevertheless, some limitations for the clinical application of Rvs. Production of Rvs is deficient in certain diseases related to chronic, unresolved inflammation. Besides, it is difficult to produce Rvs from synthetic pathways. At present, stable analogs of Rvs are being developed, whereas delivery methods are also being improved. Thus, more work should be done to achieve clinical application of Rvs for the treatment of neurological diseases in the future.

## Author Contributions

KL conceived the topic and designed the outline of this review; CL drafted the manuscript; XW and SL contributed to the literature review and manuscript writing; DS and JZ prepared the figures; KL critically revised the manuscript. All listed authors have approved the submission and publication of the manuscript.

## Funding

This study was supported by grants from The First Hospital, Jilin University, Changchun, the National Natural Science Foundation (81471216, 81771299) as well as from the Swedish Research Council (project with 2015-03005).

## Conflict of Interest

The authors declare that the research was conducted in the absence of any commercial or financial relationships that could be construed as a potential conflict of interest.
